# Delayed sub-aponeurotic fluid collections in infancy: Three cases and a review of the literature

**DOI:** 10.4103/2152-7806.66622

**Published:** 2010-07-21

**Authors:** Anthony L. Petraglia, Michael J. Moravan, Andrew H. Marky, Howard J. Silberstein

**Affiliations:** Department of Neurosurgery, University of Rochester Medical Center, Rochester, New York, USA

**Keywords:** CSF leak, fetal scalp electrodes, sub-aponeurotic fluid collection

## Abstract

**Background:**

Sub-aponeurotic fluid collections (SFCs) in the neonatal period are poorly described in the literature. We describe the occurrence, possible etiologies and treatment of sub-aponeurotic fluid collections following the neonatal period.

**Case Description:**

We present 3 cases of previously healthy children who developed soft, fluctuant, extracranial masses several weeks after birth. All 3 children were seen by a pediatric neurosurgeon after parents noticed scalp masses between 5 and 9 weeks of age. All 3 children were found to be otherwise healthy. Two of the children were born via C-section and 1 child was born vaginally. The vaginal delivery was described as difficult and utilized vacuum assist. Scalp electrodes were placed in all 3 children for intensive monitoring during labor. These children received plain skull x-rays to assess for abnormalities, and 2 of the children underwent a non-contrast brain CT scan to better characterize the fluid collection. Plain x-rays and CT scans showed no abnormalities of the skull or ventricles. In both patients who underwent a CT scan, a soft tissue prominence was noted with a Hounsfield unit similar to water. All cases resolved between 5 and 9 weeks after initial presentation, with no long-term sequelae.

**Conclusion:**

SFCs presenting after the neonatal period are usually associated with benign soft tissue swellings. Use of fetal scalp electrodes has been shown to cause cerebrospinal fluid (CSF) leakage in the neonatal period and may result in delayed SFC. This condition is benign, and the recommended course of treatment is conservative management.

## INTRODUCTION

Sub-aponeurotic fluid collections (SFCs) of CSF have been previously described; however, the number of cases in the literature remains small. Scalp swelling in the neonatal period is common, and causes can include cephalohematoma, caput succedeneum, sub-aponeurotic hemorrhage and SFCs.[5,9] The natural history of SFCs compared to cephalohematoma and caput succedeneum differs significantly and is important to recognize. SFCs are usually clinically obvious, are thought to be the result of CSF leakage, are often reported in the weeks following delivery, and resolve spontaneously with conservative management.[5,9] Cephalohematoma is a collection of blood in the subperiosteal space, often slow to resolve and frequently due to traumatic birth. Caput succedeneum is a subcutaneous blood collection present at birth, resolving over a few days.[5]

## CASE REPORT

### Clinical presentation

Three children, born at term, with scalp swelling were referred to the neurosurgery department for further evaluation. All 3 children were found to have sub-aponeurotic fluid collections. The clinical and imaging findings, method of treatment and outcome were recorded. Obstetric history and neonatal history were obtained retrospectively.

Clinical features are displayed in Table 1. All 3 cases presented in infancy between 5 and 9 weeks following delivery. One had vacuum-assisted delivery after labor failed to progress and there was found to be no descent of the fetal vertex. The other two were delivered via C-section without complication. All 3 children had fetal scalp electrodes placed for intensive monitoring during labor. All children were found to be otherwise healthy following delivery and in the early neonatal period.

**Table 1:** Clinical characteristics of the 3 cases of sub-aponeurotic fluid collection

Patient	Gestation (weeks)	Delivery mode	Fetal scalp electrodes (Y/N)	Age at presentation (weeks)	Management	Time for resolution of scalp swelling (weeks)
1	39	Vaginal-vacuum assist	Y	9	Conservative	5
2	40	Cesarean section	Y	5	Conservative	6
3	39	Cesarean section	Y	5	Conservative	9

**Figure 1(a-d):**
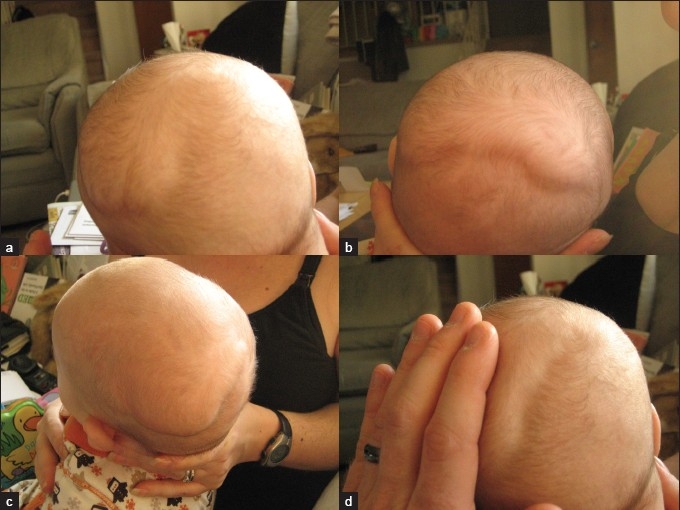
Images of patient with sub-aponeurotic CSF collection demonstrating a soft, non-tender and fluctuant scalp mass

Upon clinical examination by a pediatric neurosurgeon at URMC, all 3 children were found to be awake and alert, no abnormalities of the skull were found on palpation and all children were reaching appropriate developmental milestones. Exams revealed non-tender, soft compressible and fluctuant scalp masses in all patients [Figures 1a–d]. These scalp masses were also noted to cross suture lines. No other gross abnormalities of the scalp were noted, and none of the patients were otherwise symptomatic.

**Figure 2:**
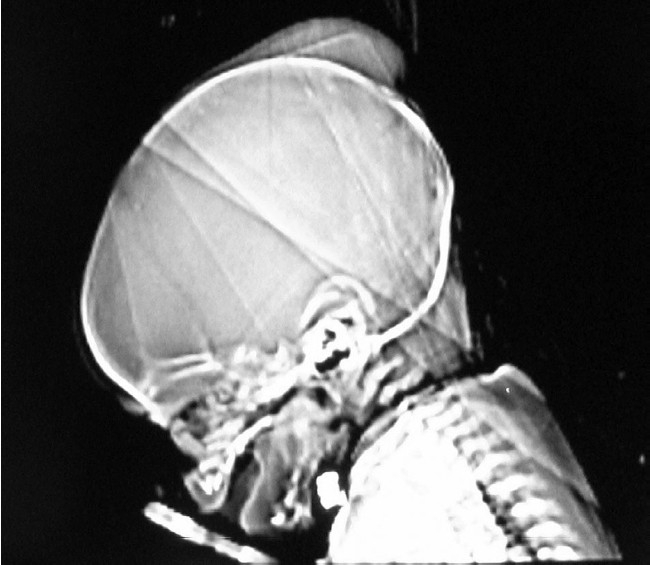
Plain radiograph demonstrating well-circumscribed smooth mass overlying the skull, consistent with sub-aponeurotic CSF collection. There were no signs of skull fracture, abnormal areas of lucency or sclerosis

Skull radiographs in 2 of the 3 patients demonstrated well-circumscribed smooth masses overlying the skull. In the third case, anteroposterior (AP) and lateral views of the skull showed no soft tissue abnormality, despite its gross appearance on physical exam. In all 3 patients, skull radiographs showed no signs of skull fracture, abnormal areas of lucency or sclerosis [Figure 2].

**Figure 3:**
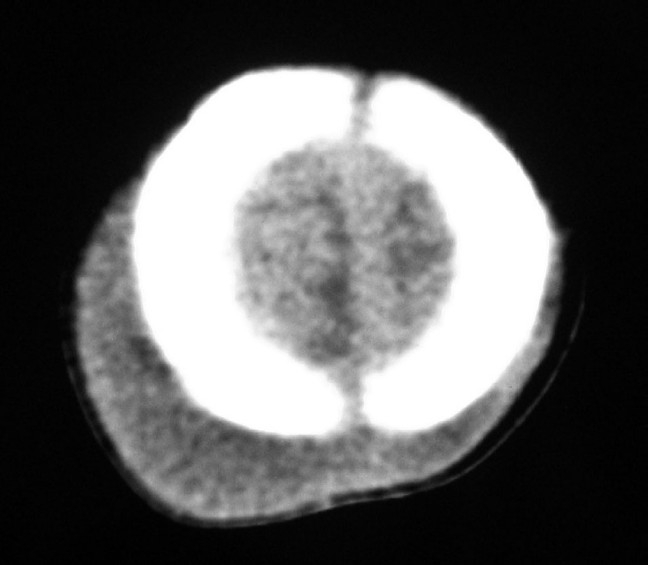
Non-contrast head CT of infant demonstrating soft tissue fluid collection. The Hounsfield units were consistent with water/ CSF. No fractures were identified

Computed tomography (CT) scans of the brain without contrast were obtained for 2 of the patients. The CT scans better characterized the soft tissue swellings in both patients and confirmed the skull radiograph findings [Figure 3]. In both patients who underwent a CT scan, the ventricles, cisterns and cerebrospinal fluid (CSF) spaces were normal in size, shape and position. The fluid collections were noted to have Hounsfield units similar to water and not hemorrhage. There was no evidence of intracranial hemorrhage or mass effect. All 3 patients were treated conservatively, and the sub-aponeurotic fluid collections resolved between 5 and 9 weeks following their initial appearance.

## DISCUSSION

The etiology of SFCs remains largely uncertain.[5] SFC is an important differential diagnosis in young infants with skull masses, and previous studies suggest that SFC, like sub-aponeurotic hemorrhage (SH), may be linked to birth trauma, as is commonly seen with prolonged vacuum-assisted delivery.[9] In addition to traumatic birth, disruption of scalp lymphatic drainage, venous drainage; and CSF leakage are likely contributors.[9] In a small case series of SFC, Schoberer *et al*.[9] proposed that while major bone fractures were not present in their patients, microfractures may have been present and disrupted emissary and diploic veins that connect intracranial venous sinuses with superficial veins of the scalp. However, analysis of several aspirates from their patients showed that CSF was found in all samples. The authors reported that the origin of this CSF was uncertain.

Based on the imaging data and clinical exam, the scalp swellings in our patients were consistent with CSF. Our data neither support nor disprove previous hypotheses that SFCs are related to traumatic labor,[5] as only 1 of our 3 children was delivered with vacuum extraction in what was described by the attending obstetrician as a difficult delivery. However, all 3 children were monitored with fetal scalp electrodes (FSEs). FSE-monitoring has previously been associated with serious sequelae, including CSF leakage, meningitis and even death from complications.[3,6,12] While Schoberer *et al*.[9] found CSF in all aspirates from their patients, they were unable to ascertain the origin of the CSF. It has been hypothesized that the thin skull bones of the baby allow piercing of the dura and arachnoid by the electrode, even when the electrode is applied away from fontanelles or suture lines.[10] This may explain the source of CSF leakage sometimes found in neonates who had FSEs used during their delivery. Although potential perinatal improvement frequently outweighs the cost of fetal monitoring,[1] it is imperative that physicians be aware of the potential complications it may cause, and consider this as a possible etiology of SFC following the birth of a child in which FSEs were used.

The unique aspect of SFC in this case series is the use of FSEs in all 3 patients. Past studies have identified FSEs as a known cause of CSF leakage in the neonate,[4,7,10,11] and CSF leakage as a cause of SFC.[5,9] The unusual aspect of these patients is that in contrast to previous case reports, neonates who suffered a puncture wound from a FSE and subsequent CSF leakage, had CSF leaks large enough to be identified immediately after birth. In this series of patients, the CSF leak was not identified immediately after birth, and SFC was not diagnosed until several weeks following birth. Although CSF cranial leaks in neonates are rare,[8] we propose the hypothesis that the presentation of patients with SFC caused by CSF leakage, possibly as a result of FSE-monitoring, may be delayed after birth, as is suggested in the cases of our 3 patients. In short, we believe that delayed CSF accumulation in infants may be caused by the use of FSEs, which cause a small puncture wound allowing slow gradual leakage of CSF and subsequent SFC in the weeks following birth. Fetal scalp electrodes are used commonly, and many small subgaleal CSF collections may not even come to the attention of practitioners. One of the limitations of this study is the small patient sample size. While the scalp electrode is a common thread in these 3 cases, we can only speculate about this association. A larger series of patients and further studies will be needed to determine whether or not the fetal scalp electrodes and subgaleal CSF collections are related.

Although imaging findings can aid in differentiating these CSF collections from other etiologies of scalp swelling, the diagnosis is primarily a clinical one. These CSF collections are fluctuant and do not tend to cause periosteal ridging as seen with cephalohematomas. Aspiration of these lesions has proven unnecessary and carries the potential risk of infection.[2,5,9] Schoberer *et al*.[9] in a study in which 3 out of 5 patients with SFC underwent therapeutic punctures reported that the lesions refilled in all 5 patients but later resolved spontaneously. Hopkins *et al*.,[5] who published the first case series describing SFC, reported that 1 of their 7 patients was treated with needle aspiration on two occasions. The fluid was found to be sterile, and no biochemical analysis was performed. Once again, the collection resolved spontaneously in the weeks following presentation, and was not aided by needle aspiration. We have not seen a case of subgaleal CSF collection persist beyond 2 months; however, if a collection were to persist beyond 3 months, we would recommend an MRI to evaluate for a persistent conduit for CSF egress into the subgaleal space. Further management or exploration would depend on such imaging findings.

The authors acknowledge that this phenomenon is a likely common presentation in the pediatric neurosurgery clinic; however, with only 12 cases of SFC described in the literature, all of which are from European countries, it is important that clinicians are aware of this and are prepared to manage patients appropriately. Taking into account previous published accounts of SFC, and our own experiences, management of sub-aponeurotic fluid collections should be conservative and these collections do not need to be aspirated, as most will spontaneously resolve.
